# A Magnetic Nanoparticle Based Enzyme-Linked Immunosorbent Assay for Sensitive Quantification of Zearalenone in Cereal and Feed Samples

**DOI:** 10.3390/toxins7104216

**Published:** 2015-10-20

**Authors:** Xian Zhang, Xin Wang, Mengjiao Sun, Xiaofeng Zhang, Houhui Song, Yaxian Yan, Jianhe Sun, Xiaoliang Li, Weihuan Fang

**Affiliations:** 1 Institute of Preventive Veterinary Medicine and Zhejiang Provincial Key Laboratory of Preventive Veterinary Medicine, Zhejiang University, 388 Yuhangtang Road, Hangzhou 310058, China; E-Mails: zhangian073@163.com (X.Z.); wangxin20095017@126.com (X.W.); sunmengjiao265@126.com (M.S.); xlli@zju.edu.cn (X.L.); 2 Food Safety Key Laboratory of Zhejiang Province, Zhejiang Entry-Exit Inspection and Quarantine Bureau, 126 Fuchun Road, Hangzhou 310012, China; E-Mail: zxf@ziq.gov.cn; 3 College of Animal Science and Technology, Zhejiang A&F University, 88 Huanbei Road, Lin’an 311300, China; E-Mail: songhh@zafu.edu.cn; 4 School of Agriculture and Biology, Shanghai Jiaotong University, Shanghai 200240, China; E-Mails: yanyaxian@sjtu.edu.cn (Y.Y.); sunjhe@sjtu.edu.cn (J.S.)

**Keywords:** zearalenone, immunoassay, magnetic nanoparticles, biotin-streptavidin, quantification

## Abstract

A novel enzyme-linked immunosorbent assay based on magnetic nanoparticles and biotin/streptavidin-HRP (MNP-bsELISA) was developed for rapid and sensitive detection of zearalenone (ZEN). The detection signal was enhanced and the sensitivity of the assay was improved by combined use of antibody-conjugated magnetic nanoparticles and biotin-streptavidin system. Under the optimized conditions, the regression equation for quantification of ZEN was *y* = −0.4287*x* + 0.3132 (*R*^2^ = 0.9904). The working range was 0.07–2.41 ng/mL. The detection limit was 0.04 ng/mL and IC_50_ was 0.37 ng/mL. The recovery rates of intra-assay and inter-assay ranged from 92.8%–111.9% and 91.7%–114.5%, respectively, in spiked corn samples. Coefficients of variation were less than 10% in both cases. Parallel analysis of cereal and feed samples showed good correlation between MNP-bsELISA and liquid chromatograph-tandem mass spectrometry (*R*^2^ = 0.9283). We conclude that this method is suitable for rapid detection of zearalenone in cereal and feed samples in relevant laboratories.

## 1. Introduction

Zearalenone (ZEN), a mycotoxin, is produced by Fusarium species growing on grains, mainly corn and hay exposed to high moisture during storage, such as *Fusarium graminearum* and *Fusarium roseum* [1,2]. Over time, the toxicity of ZEN in food and animal feed has been widely recognized. ZEN can cause hyperestrogenism in livestock, leading to diseases and harmful effects associated with the reproductive system such as abortion and stillbirth [3,4,5]. Products contaminated by ZEN compromise food safety and threaten human health. Babies and children are more vulnerable to the effects of ZEN than adults [6]. Therefore, the Joint Food and Agriculture Organization (FAO)/World Health Organization (WHO) Expert Committee on Food Additives (JECFA) has established the provisional maximum tolerable daily intake levels of 0.5 μg/kg of body weight per day for ZEN [7].

The detection methods of mycotoxins mainly consist of chromatography and immunology-based analytical approaches. Chromatographic methods, including thin-layer chromatography [8], liquid chromatography-tandem mass spectrometry (LC-MS/MS) [9,10], and high-performance liquid chromatography [11,12], are sensitive and produce reliable results. However, the complex preparation steps, expensive equipment, and time-consuming procedures make such methods unsuitable for routine work in many laboratories and other locations, such as farms or factories. Compared with chromatographic methods, a number of immunoassays have been developed for mycotoxins detection in agricultural products with the advantages of rapid and cost effective, such as enzyme-linked immunosorbent assay (ELISA) [13,14,15], colloidal gold lateral flow immunoassay [16,17,18], electrochemical immunosensor assay (EIA) [19,20] and fluorescent linked immunosorbant assays (FLISAs) [21,22,23]. In case of mycotoxins, sensitive, accurate and rapid analytical methods are still needed, thus requiring new strategies for signal enhancement and time-saving procedures.

Magnetic nanoparticles are recently used in assays of biomedical and food-safety fields with the advantages of uniform diameters and even distribution in solution [24,25,26]. Complexes between the nanoparticles and antibodies are formed by covalent immobilization. The immobilized particles can bind with the target antigens in solution and are rapidly separated by a magnetic field [27,28,29]. This technology has the advantages of liquid-phase immunological reactions, reduced detection time and improved sensitivity [30]. Biotin-streptavidin coupling is one of the best characterized systems for signal amplification [31,32,33].

**Figure 1:**
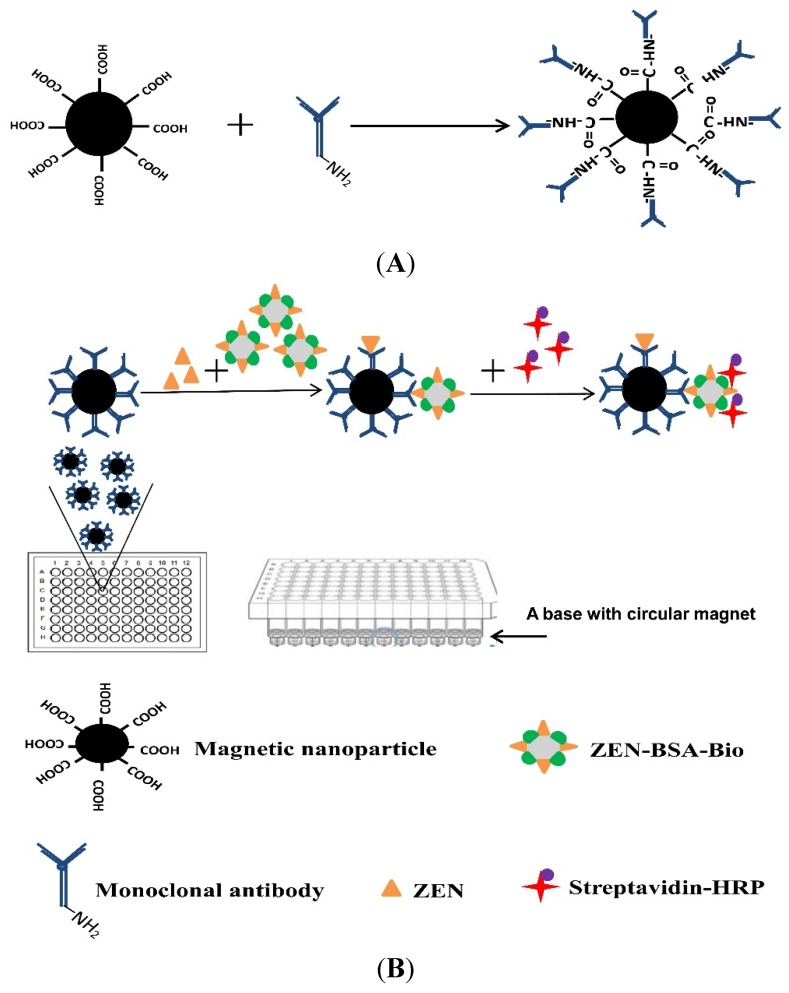
Schematic diagrams of the preparation of the immunomagnetic nanoparticles cross-linked with anti-zearalenone monoclonal antibody (MNP-anti-ZEN) (**A**) and MNP-bsELISA (**B**).

Here, we report a novel ELISA strategy (MNP-bsELISA)) for sensitive detection of ZEN in cereal and feed samples. A schematic diagram of the MNP-bsELISA is shown in Figure 1. In this assay, monoclonal antibody coated magnetic nanoparticles (MNP-Anti-ZEN) and biotinylated ZEN-BSA conjugate (ZEN-BSA-Biotin) were used and the detected format is based on the indirect competitive enzyme-linked immunosorbent assay (ic-ELISA). The test is completed in a 96-well plate by using a base with circular magnet. This assay is suitable for high throughput detection and proves to be more sensitive and less time-consuming than the same antibody based conventional ELISA in an earlier report [34].

## 2. Results and Discussion

### 2.1. Identification of ZEN-BSA Conjugate and ZEN-BSA-Biotin

**Figure 2:**
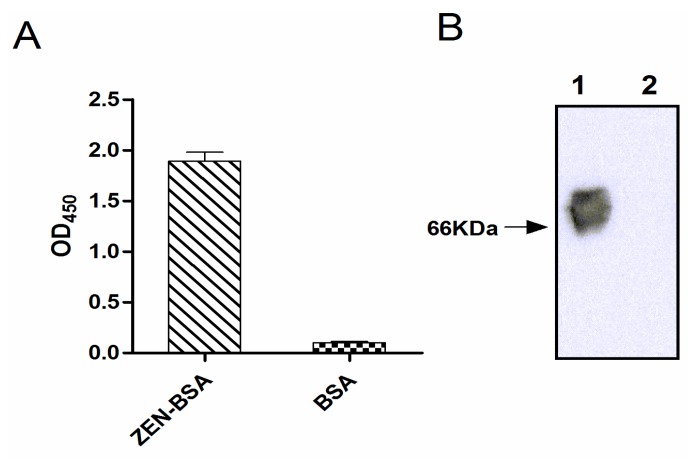
Identification of conjugation of zearalenone with bovine serum albumin (ZEN-BSA) by indirect ELISA (**A**) and Western blotting (**B**). The molecular weight of BSA is about 66 KDa. Lane 1: ZEN-BSA, Lane 2: BSA.

Indirect ELISA and Western blotting indicated that ZEN was successfully conjugated to the carrier protein BSA (Figure 2). There was no signal from the BSA control. For quantitation of the extent of biotin incorporation into ZEN-BSA, the dye HABA was used for colorimetric analysis of the colored complex with avidin displaced by biotin. We obtained a biotinylation level of 3.7:1 (ZEN-BSA: Biotin).

### 2.2. Identification of Anti-ZEN Immunomagnetic Nanoparticles

**Figure 3:**
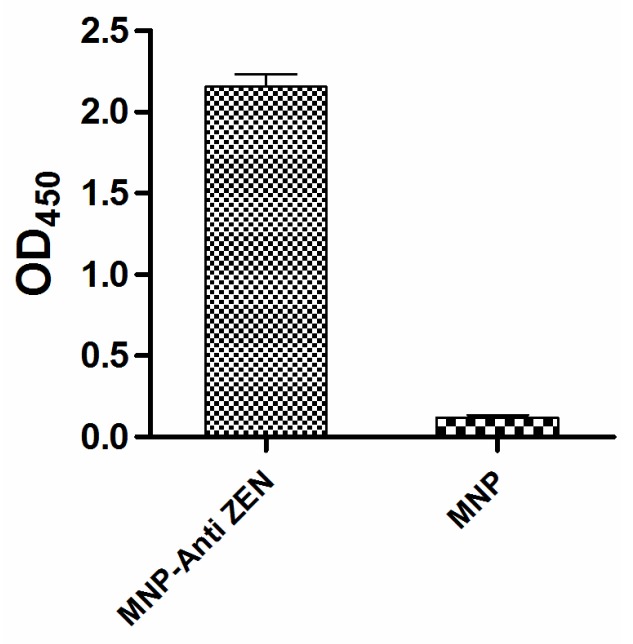
Identification of the immunomagnetic nanoparticles cross-linked with anti-zearalenone monoclonal antibody (MNP-anti-ZEN) by indirect MNP-bsELISA.

The anti-ZEN mAb was purified (4 mg/mL) and titrated with indirect ELISA. Indirect ELISA based on biotin-streptavidin-HRP system showed that anti-ZEN mAb was successfully conjugated to the magnetic nanoparticles (Figure 3): 20 μg per mg magnetic nanoparticles as analyzed by BCA method on the protein concentration of the reaction solution before and after coupling.

### 2.3. Optimization of Indirect Competitive MNP-bsELISA for Quantitation of ZEN

**Figure 4:**
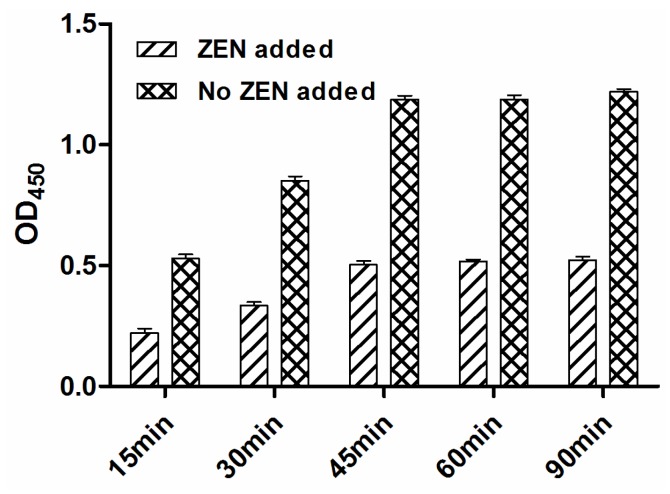
Determination of suitable incubation time by indirect ELISA with (+ZEN) or without (−ZEN) addition of zearalenone.

In MNP-bsELISA, the amounts of immunomagnetic nanoparticles and ZEN-BSA-Biotin would significantly affect the assay performance. Higher concentrations of nanoparticles could cause high background and poor sensitivity. Checkerboard titration showed that the optimum dilution of the nanoparticles was 1:100 and the optimal concentration of the ZEN-BSA-Biotin was 0.0025 μg/mL. Strep-HRP was optimal at 1:2000 (or 0.5 μg/mL), and incubation time optimal at 45 min (Figure 4).

### 2.4. Specificity Study

Using the optimized MNP-bsELISA method, the cross-reactivities with the ZEN analogues (α-zearalanol, zearalanone, α-zearalenol, β-zearalenol and β-zearalanol) were 27%, 15%, 11%, 0.7% and 0.4%, respectively. The same antibody (mAb 2C9) was found to have 16% cross-reactivity on average with other ZEN analogues in ic-ELISA (the cross-reactivities with α-zearalanol, zearalanone and β-zearalanol were 32%, 17% and 0.1%). Others also found cross-reactivity with ZEN analogues [35,36,37]. High structural similarity between ZEN and its analogues was the apparent reason. Such level of cross-reactivity in this method was relatively low and can be accepted. No cross-reactivity (<0.01%) was observed with other mycotoxins (AFB1, FB1, DON and OTA) which usually occur together in cereal and feed samples. These results indicated that this novel method has good specificity.

### 2.5. Calibration Curve and Matrix Interference Analysis

**Figure 5:**
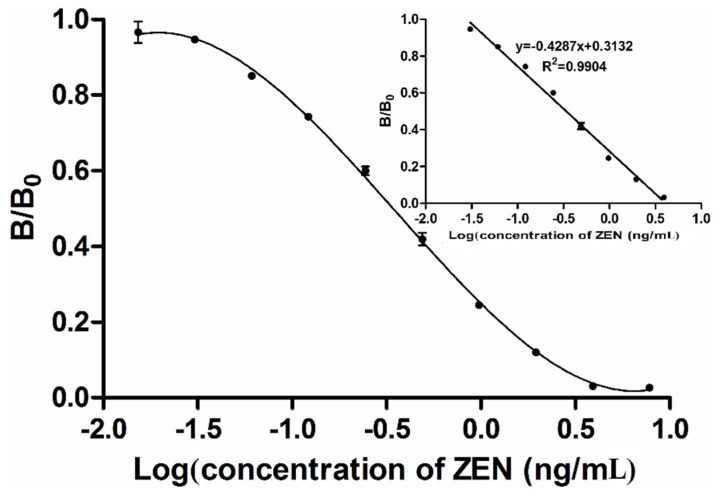
Tri-parametric curve fitting of log concentration of zearalenone *vs.* inhibition index by MNP-bsELISA. The insert shows the standard curve for quantification of zearalenone.

A calibration curve of the MNP-bsELISA was prepared under the optimized conditions and calculated with the GraphPad5 software (GraphPad Software, La Jolla, CA, USA) (Figure 5). The linear range of detection was calculated as the concentration of ZEN leading to 20%–85% inhibition according to the previous study [38]. The limit of detection (LOD) was calculated as the average signal corresponding to three standard deviations from the signals of ZEN-free corn samples (*n* = 5) [39,40]. The regression equation for quantification of ZEN was *y* = −0.4287*x* + 0.3132 (*R*^2^ = 0.9904). The working range was 0.7–24.1 μg/kg. The detection limit was 0.4 μg/kg, and IC_50_ (50% inhibition of the maximum OD value) was 3.7 μg/kg. A conventional indirect competitive ELISA (ic-ELISA) based on this antibody was also established in our laboratory [34], the regression equation was *y* = −0.3909*x* + 0.5367 (*R*^2^ = 0.9940) with the working range from 0.21–9.76 ng/mL, IC_50_ at 1.24 ng/mL and limit of detection at 0.12 ng/mL. Table 1 shows that the MNP-bsELISA is more sensitive and faster than ic-ELISA using the same mAb 2C9 [34] and has an advantage of lower detection limit than previous reported methods such as ELISAs, fluorescence assay and array immunoassay [39,40,41,42]. Assays based on immunosensor [35] require expensive equipment, time-consuming procedures and skilled operators and are not suitable for rapid detection of toxins, although it has greater sensitivity. In the MNP-bsELISA, the signal was enhanced and the sensitivity was improved by combining using of magnetic nanoparticles and biotin-streptavidin system. With increases in binding and competition efficiency, the reaction time also decreased.

**Figure 6:**
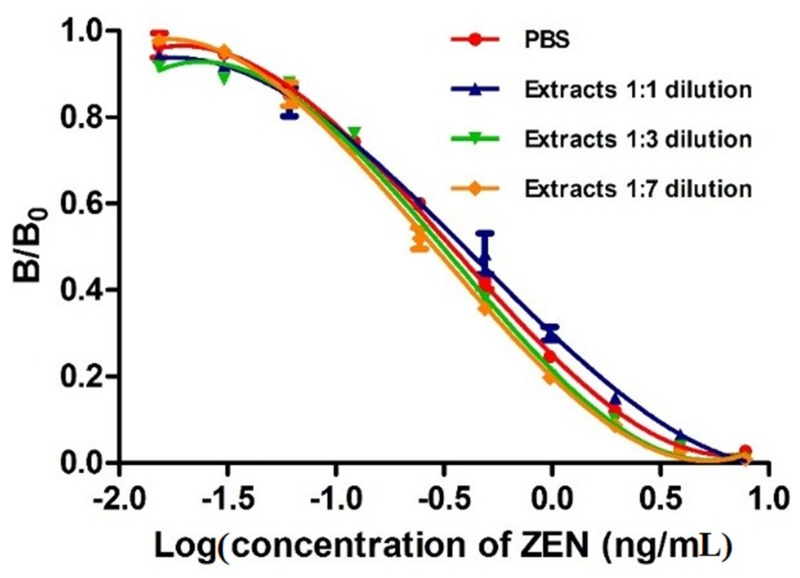
Analysis of matrix interferences by different dilutions, with PBS, of corn extracts spiked with different concentrations of zearalenone by MNP-bsELISA.

Methanol in the extracted solution buffer and the components in the sample extracts, including proteins and vitamins, are known to affect the immuno-reagents and immune-reactions [43,44]. In this study, matrix interference was determined by comparing a calibration curve prepared in PBS with those obtained in serial diluted ZEN-free corn sample extracts. Figure 6 shows that all dilutions of the extracts with PBS reducing the matrix interference. Because these curves were all superimposed, we suggest that 1:1 dilution is enough to minimize the matrix effect. Matrix interference was often excluded by substantial dilution of the sample prior to analysis according to previous studies [45,46]. However, higher dilution would cause proportional reduction of the assay sensitivity. Therefore, a 1:1 dilution was selected for recovery test and detection of ZEN in cereal and feed samples in this study.

**Table 1:** Comparison of different analytical methods for detection of zearalenone.

Methods	Detection Time	Limit of Detection	IC_50_	Working Range	Ref.
DC-ELISA	1h	0.15 ng/mL	1.13 ng/mL	41.0–909.8 μg/kg	[40]
ic-ELISA	2h	0.8 ng/mL	-	0.8–150 ng/mL	[41]
Immunosensor assay	-	0.007 ng/mL	-	0.019–0.422 ng/mL	[35]
Fluorescence assay	-	137 μg/kg	-	150–1000 μg/kg	[39]
Array Immunoassay	1.5	0.51 ng/mL	2.1 ng/mL	0.73–6.8 ng/mL	[42]
mAb 2C9 based ic-ELISA	2 h	0.12 ng/mL	1.24ng/mL	0.21–9.76 ng/mL	[34]
mAb 2C9 based MNP-bsELISA	1.5	0.04 ng/mL	0.37 ng/mL	0.07–2.41 ng/mL	This study

- not mentioned.

### 2.6. Recovery Rates of ZEN in Spiked Corn Samples and Detection of ZEN in Natural Samples

A ground corn sample without ZEN contamination was spiked with different concentrations of ZEN, and the recovery rates were determined by MNP-bsELISA. Table 2 shows that the recovery rates of intra-assay and inter-assay ranged from 92.8% ± 6.9% to 111.9% ± 6.7% and 91.7% ± 7.3 to 114.5% ± 6.4%, respectively, in spiked corn samples. The coefficients of variation (CVs) were 3.1%–7.5% for intraday comparisons and 4.6%–8.7% for interday comparisons. These results indicate that the method has good recovery from spiked samples with high precision.

**Table 2:** Recovery and coefficient of variances from corn samples spiked with different levels of zearalenone by MNP-bsELISA.

Samples	Spiked Level (μg/kg)	Inter-Assay ^a^			
		*n*	Measured (μg/kg)	Recovery (%)	CV ^b^ (%)
1	1.25	3	1.18 ± 0.04	94.5 ± 2.9	3.1
2	2.5	3	2.32 ± 0.18	92.8 ± 6.9	7.5
3	5	3	5.42 ± 0.26	108.4 ± 5.2	4.8
4	10	3	11.19 ± 0.67	111.9 ± 6.7	6.1
5	20	3	21.43 ± 1.13	107.2 ± 5.6	5.3

^a^ Inter-assay variation was determined each spiked level on 3 days; ^b^ Coefficient of variation.

**Figure 7:**
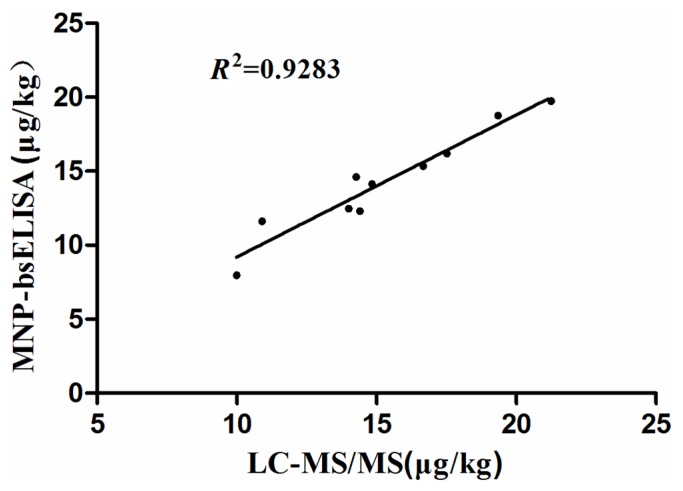
Correlation of results obtained by both MNP-bsELISA and LC-MS/MS for zearalenone detection in natural cereal and feed samples.

The natural samples were analyzed using both the MNP-bsELISA and LC-MS/MS. The results are shown in Table 3 (only positive samples are listed). Given that the detection limit of LC-MS/MS was 5 ug/kg, levels of ZEN below this could not be detected, but could be detected by MNP-bsELISA. Ten samples were subjected to quantitative analysis by both methods. The relationship between MNP-bsELISA and LC-MS/MS results for ZEN in cereal and feed samples was assessed by regression analysis, MNP-bsELISA = 0.395 + 0.9599LC-MS/MS (*R*^2^ = 0.9283), indicating a good agreement between the two methods (Figure 7). These results demonstrate that the developed MNP-bsELISA can be used for ZEN detection in cereal and feed samples.

**Table 3:** Comparison of MNP-bsELISA with LC-MS/MS for detection of zearalenone in cereal and feedstuff samples.

Samples	MNP-bsELISA (μg/kg), Mean ± SD ^a^	LC-MS/MS (μg/kg), Mean ± SD
Corn 1	7.96 ± 0.49	9.99 ± 0.08
Corn 2	12.47 ± 0.66	14.01 ± 0.45
Corn 3	14.61 ± 0.37	14.27 ± 0.15
Corn 4	11.61 ± 0.46	10.91 ± 0.28
Corn 5	3.02 ± 0.19	- ^b^
Wheat 1	15.34 ± 0.39	16.67 ± 0.05
Wheat 2	3.76 ± 0.21	- ^b^
Wheat 3	12.31 ± 0.26	14.41 ± 0.35
Feedstuff 1	19.73 ± 1.69	21.25 ± 0.11
Feedstuff 2	16.19 ± 0.71	17.52 ± 0.29
Feedstuff 3	14.13 ± 0.77	14.84 ± 0.28
Feedstuff 4	18.77 ± 1.19	19.35 ± 0.16

^a^ SD, standard deviation (*n* = 3). ^b^ -, not detected.

In conclusion, a novel enzyme-linked immunosorbent assay to determine ZEN levels in cereal and feed samples was developed using a system that involves magnetic nanoparticles and biotin/streptavidin-HRP (MNP-bsELISA). The method was more sensitive and rapid than conventional ELISA and had good correlation with the LC-MS/MS in quantifying zearalenone in cereal and feed samples. As a new strategy for the detection of low-molecular-weight analytes, this novel method is suitable for rapid detection of zearalenone in cereal and feed samples in relevant laboratories once it is optimized as a kit and could also be easily extended to rapid detection of other mycotoxins and biological analytes.

## 3. Experimental Section

### 3.1. Chemicals and Reagents

Zearalenone (ZEN), fumonisin B1 (FB1), aflatoxinB1 (AFB1), ochratoxin A (OTA), deoxynivalenol (DON), carboxymethoxylamine hemihydrochloride (CMO), bovine serum albumin (BSA), *N,N*′-dicyclohexylcarbodiimide (DCC), *N*-hydroxy-succinimide (NHS), 2-Morpholinoethanesulfonic acid (MES) and *N*,*N*-Dimethylformamide (DMF) were purchased from the Sigma Chemical Co. (St. Louis, MO, USA). ZEN analogues (α-zearalanol, α-zearalenol, β-zearalanol, β-zearalenol, and zearalanone) were purchased from UcallM Biotechnology Co. (Wuxi, Jiangsu, China). A hybridoma cell line secreting monoclonal antibodies against ZEN (mAb 2C9) was prepared in our laboratory. EZ-Link sulfo-NHS-LC-Biotinylation Kit (21435) was purchased from Thermo Fisher Scientific (Wyman Street, Waltham, MA, USA), Streptavidin-horseradish peroxidase (Streptavidin-HRP) was obtained from Anaspec (Fremont, CA, USA). Magnetic nanopaticles M-270 carboxylic acid (14305D) was obtained from Invitrogen (Carlsbad, CA, USA). Other reagents of analytical grade, including 3,3,5,5-tetramethylbenzidine (TMB, as ELISA substrate), were purchased from Sinopharm Chemical Reagent Co. Ltd. (Shanghai, China). Both the ZEN-free and naturally contaminated cereal and feed samples were provided by the Zhejiang Entry-Exit Inspection and Quarantine Bureau (Hangzhou, Zhejiang, China).

### 3.2. Equipment

The 96-well plates for microsphere-based ELISA were purchased from Tianlong industry (Haimen, Zhejiang, China); the 37 °C incubator from Thermo Scientific (Waltham, MA, USA); the horizontal shaker (Vortex 4 basic) from IKA (Staufen, Germany); the magnetic separator (MS-12) from Bangs laboratories (Fishers, IN, USA); Spectra Max M2 micro-plate reader (Molecular Devices, Sunnyvale, CA, USA) was used for absorbance measurement; the ultraperformance liquid chromatography (UPLC) system and UPLC BEH C18 column were supplied by Waters (Milford, MA, USA); the QTrap MS/MS system was obtained from Applied Biosystems (Foster City, CA, USA).

### 3.3. Synthesis of the ZEN-BSA Conjugate

The ZEN-BSA conjugate was synthetized as described previously with slight modifications [47,48]. Zearalenone (1.0 mg) and carboxymethoxylamine hemihydrochloride (2.0 mg) were dissolved in 1.0 mL pyridine and stirred for 24 h at room temperature (RT). The reaction mixture was then dried under vacuum, and the residue was dissolved in 4 mL of distilled water (pH adjusted to 8.0 with sodium hydroxide). Free zearalenone was removed from the water phase by three partitions with benzene (3.0 mL). The reaction mixture in the aqueous phase was then precipitated by addition of HCI (pH 3.0) and extracted four times with 10 mL of ethyl acetate. The extract was dehydrated over anhydrous sodium sulfate and dried under vacuum. The residue was dissolved in 0.4 mL of anhydrous tetrahydrofuran and added to 6.0 mg DCC and 3.0 mg NHS. The mixture was allowed to react for 1 h by shaking gently at RT, and then centrifuged at 2500× *g* for 30 min. The precipitate was discarded and the aquaous phase was dried under vacuum. The final derivative was dissolved in 2.0 mL DMF. BSA (20 mg) was dissolved in 2.0 mL of 130 mM phosphate buffer (pH 7.4). The activated-ZEN was added drop-wise to the BSA solution. Reaction was allowed to proceed for 1 h by gently shaking at RT. The solution was centrifuged to discard the precipitates. Finally, the supernatant was dialyzed against 0.01 mol/L PBS (pH 7.4) at 4 °C for 72 h to remove the residual free ZEN and DMF. The ZEN-BSA conjugate was confirmed by reacting with anti-ZEN mAb using indirect ELISA and Western blotting. BSA was used as control. The final product was stored at −20 °C for later use. 

### 3.4. Biotinylation of ZEN-BSA and Identification

Biotinylated ZEN-BSA was synthetized as described by the supplier (Thermo scientific; 21435). ZEN-BSA was dissolved in reaction buffer (0.01 M PBS, pH 7.4), and mixed with sulfo-NHS-LC-biotin by slow addition. The solution was allowed to react for 2 h at RT. The biotinylated derivative was purified by gel filtration on Zeba™ Spin Desalting Columns (Thermo scientific, Waltham, MA, USA; 89891). The level of biotinylation was measured by HABA (4′-hydroxyazobenzene-2-carboxylic acid) competition assay according to previous studies [49,50].

### 3.5. Preparation of Immunomagnetic Nanoparticles

The immunomagetic nanoparticles were synthetized as described by the supplier (Invitrogen, Waltham, MA, USA; 14305D). The magnetic nanoparticle stock solution (100 μL) was transferred to a new Eppendorf tube which was then placed in a magnetic field to separate the nanoparticles from the storage solution. The nanoparticles were washed twice with coating buffer (25 mM MES buffer containing 0.05% Tween 20 (*v*/*v*), pH 5.0) (This buffer was prepared using the reagent mentioned in Section 3.1) before covalent coupling with the anti-ZEN antibody. EDC and NHS solutions (each at a concentration of 50 mg/mL) were prepared in coating buffer immediately before use. A volume of 50 μL each of EDC and NHS solutions were added to the washed magnetic nanoparticles and incubated with slow rotation at RT for 30 min. The tube containing the reaction mixtures was placed in a magnetic field to remove the supernatant and washed twice with coating buffer. The purified anti-ZEN monoclonal antibody (100 μg) in coating buffer (100 μL) was added to the activated nanoparticles and incubated for at least 30 min at RT, or 2 h at 4 °C with slow tilting rotation. The antibody-coated nanoparticles were washed with washing buffer (0.01 M PBS containing 0.02% Tween 20 (*v*/*v*), pH 7.4) (This buffer was prepared using the reagent mentioned in Section 3.1). In order to quench the non-reacted activated carboxylic acid groups, the coated nanoparticles were incubated with quenching buffer (50 mM Tris containing 0.05% Tween 20 [*v*/*v*], pH 7.4) (This buffer was prepared using the reagent mentioned in Section 3.1) for 15 min at RT with slow rotation. The antibody-coated magnetic nanoparticles (MNP-anti-ZEN) (Figure 1A) were then separated from the washing buffer and resuspended in storage buffer (washing buffer containing 0.5% BSA (*v*/*v*), pH 7.4) (This buffer was prepared using the reagent mentioned in Section 3.1). Conjugation was confirmed by indirect ELISA using biotinylated ZEN-BSA and Streptavidin-HRP. Coating efficiency was measured by analyzing the antibody concentration before and after coupling using the BCA (bicinchoninic acid) protein assay.

### 3.6. Optimization of the MNP-bsELISA

Dilutions of MNP-Anti ZEN, concentrations of the ZEN-BSA-biotin and Strep-HRP were optimized by checkerboard titration design with an OD_450_ value of about 1.0 in MNP-bsELISA. Optimization conditions included dilutions of MNP-anti ZEN from 1:25, 1:50, 1:100 to 1:200; ZEN-BSA-biotin with six 2-fold dilutions from 0.01 μg/mL; and Strep-HRP from 1:1000, 1:2000, 1:4000, 1:8000 to 1:16,000. Incubation times for the competition reaction were 15, 30, 45, 60, 75 and 90 min.

### 3.7. Development of Indirect Competitive MNP-bsELISA

MNP-Anti ZEN (10 μL) diluted with storage buffer, 70 μL ZEN-BSA-biotin solution and 70 μL of ZEN standard solution at different concentrations (serial 2-fold dilution from 7.8–0.015 ng/mL, and zero control) were added to the 96-well plate, each concentration in triplicate wells. The plate was shaken at 1000 rpm for 45 min at 37 °C and then placed on a magnetic base to precipitate the nanoparticles. The plate was washed three times with washing buffer, each followed by magnetic separation. Strep-HRP (100 μL, 0.5 μg/mL) was added and the plate was further subjected to shaking incubation at 37 °C for 45 min. The inmunomagnetic nanoparticle complexes were then separated on a magnetic base and washed three times. The substrate TMB (100 μL) was added. Stop solution (2 M H_2_SO_4_, 50 μL) was pipetted after 10 min of shaking incubation at 37 °C. OD_450_ values were determined on the Spectra Max M2 micro-plate reader. The calibration curve for MNP-bsELISA was prepared with the GraphPad 5 software: *x*-axis represents the log concentration of ZEN (ng/mL) and *y*-axis (B/B_0_), the division of OD_450_ value of standard solutions by OD_450_ at 0 ng/mL.

### 3.8. Specificity

To evaluate the specificity of MNP-bsELISA, cross-reactivity of the anti-ZEN monoclonal antibody coated nanoparticles with five ZEN analogues and several other mycotoxins (AFB1, FB1, DON and OTA) were determined. ZEN analogues include α-zearalanol, α-zearalenol, β-zearalanol, β-zearalenol, and zearalanone. The cross-relativities were determined using the individual analogue, respectively. Using MNP-bsELISA, the calibration curves of ZEN with different concentrations were established first, and then different concentrations of each analyte instead of ZEN were mixed with the same volume of the ZEN-BSA-Biotin concentration (0.0025 μg/mL), which had been optimized in the earlier procedure. The calibration curves were prepared by GraphPad 5 software. Then the IC_50_ (50% inhibition) for each analyte was calculated respectively. Cross-reactivity of the anti-ZEN monoclonal antibody coated nanoparticles with each compound was evaluated using the following formula:

Cross-reactivity (%) = (IC_50_ of ZEN)/(IC_50_ of other analytes) × 100% (1)

### 3.9. Elimination of Matrix Interference and Recovery of Spiked Samples

Matrix effect, one of the most common challenges of immunoassays of corn and feed samples analysis, was evaluated in this study by comparing several calibration curves obtained in serial diluted ZEN-free corn sample extracts with that prepared using PBS buffer alone according to previous studies [45,46].

The ZEN-free corn samples were provided by the Zhejiang Entry-Exit Inspection and Quarantine Bureau after been tested by LC-MS/MS. Before spiking and recovery tests, the ZEN-free corn samples were ground and dried by overnight incubation in a 60 °C incubator. The standard ZEN solution was spiked at levels of 1.25, 2.5, 5, 10 and 20 μg/kg to ground corn samples by dropwise addition, mixed thoroughly and allowed to stand at RT overnight. Five grams of each spiked samples was placed into a 50-mL plastic centrifuge tube. The samples were extracted with 25 mL of methanol/water (7/3, *v*/*v*) at RT by vigorous vortexing for 3 min. After centrifugation at 3500× *g* for 10 min, the supernatant samples were used in MNP-bsELISA for calculation of recovery rates. To evaluate the precision of this novel method, each sample was measured three times in one day and repeated three times on different days.

### 3.10. Detection of Natural Samples by MNP-bsELISA and LC-MS/MS

Fifty-six natural samples (including corn, wheat and feedstuff) were analyzed using MNP-bsELISA and LC-MS/MS. Each sample was tested in triplicate to calculate standard deviation. For the detection by MNP-bsELISA, the natural samples were extracted as the spiked samples and diluted 1:1 with PBS before detected. Procedures used for LC-MS/MS were as follows: before extraction, the natural samples were ground and dried overnight in a 60 °C incubator. First, ten gram of each samples was extracted with solvent mixture (40 mL, acetonitrile/water/acetic acid, 79:20:1, *v*/*v*/*v*) by vigorous shaken on a horizontal shaker for 60 min at room temperature. Second, the samples were centrifuged at 2500 *g* for 20 min after standing for 10 min. The supernatants were then mixed with the same volume of mixture (acetonitrile/water/acetic acid, 20:79:1, *v*/*v*/*v*), and passed through a 0.22 μm filter before being injected into the LC-MS/MS instrument. Quantitative LC-MS/MS results were analyzed using Analyst software (AB SCIEX, Framingham, MA, USA). The correlation between the two assays was investigated using linear regression (Microsoft Excel software, Redmond, WA, USA; 2010 version).
